# Misdiagnosis of *Clostridioides difficile* Infections by Standard-of-Care Specimen Collection and Testing among Hospitalized Adults, Louisville, Kentucky, USA, 2019–2020^1^

**DOI:** 10.3201/eid2905.221618

**Published:** 2023-05

**Authors:** Julio A. Ramirez, Frederick J. Angulo, Ruth M. Carrico, Stephen Furmanek, Senén Peña Oliva, Joann M. Zamparo, Elisa Gonzalez, Pingping Zhang, Leslie A. Wolf Parrish, Subathra Marimuthu, Michael W. Pride, Sharon Gray, Cátia S. Matos Ferreira, Forest W. Arnold, Raul E. Istúriz, Nadia Minarovic, Jennifer C. Moïsi, Luis Jodar

**Affiliations:** University of Louisville, Louisville, Kentucky, USA (J.A. Ramirez, R.M. Carrico, S. Furmanek, S. Peña Oliva, L.A. Wolf Parrish, S. Marimuthu, F.W. Arnold);; Pfizer Vaccines, Collegeville, Pennsylvania, USA (F.J. Angulo, J.M. Zamparo, E. Gonzalez, P. Zhang, S. Gray, C.S. Matos Ferreira, R.E. Istúriz, N. Minarovic, J.C. Moïsi, L. Jodar);; Pfizer, Pearl River, New York, USA (M.W. Pride)

**Keywords:** Clostridioides difficile, misdiagnosis, bacteria, infections, clinical laboratory techniques, standard-of-care specimen collection, testing, hospitalized adults, diagnosis, epidemiology, hospitalization, public health surveillance, Kentucky, United States

## Abstract

Although *Clostridioides difficile* infection (CDI) incidence is high in the United States, standard-of-care (SOC) stool collection and testing practices might result in incidence overestimation or underestimation. We conducted diarrhea surveillance among inpatients >50 years of age in Louisville, Kentucky, USA, during October 14, 2019–October 13, 2020; concurrent SOC stool collection and CDI testing occurred independently. A study CDI case was nucleic acid amplification test‒/cytotoxicity neutralization assay‒positive or nucleic acid amplification test‒positive stool in a patient with pseudomembranous colitis. Study incidence was adjusted for hospitalization share and specimen collection rate and, in a sensitivity analysis, for diarrhea cases without study testing. SOC hospitalized CDI incidence was 121/100,000 population/year; study incidence was 154/100,000 population/year and, in sensitivity analysis, 202/100,000 population/year. Of 75 SOC CDI cases, 12 (16.0%) were not study diagnosed; of 109 study CDI cases, 44 (40.4%) were not SOC diagnosed. CDI incidence estimates based on SOC CDI testing are probably underestimated.

*Clostridioides difficile* infection (CDI) is a major cause of illness and death worldwide (*1**,**2*). The Centers for Disease Control and Prevention (CDC) classifies CDI as an urgent public health threat (*3*). In the CDC Emerging Infections Program (EIP), the CDI incidence in persons >50 years of age was 255/100,000 population in 2019, and the hospitalized CDI incidence in this age group was 140/100,000 population (*4*).

CDI incidence estimates derived from public health surveillance rely on standard-of-care (SOC) stool specimen collection and CDI testing practices. Laboratory testing using only a PCR nucleic acid amplification test (NAAT), which tests for the presence of the toxin gene without testing for the presence of free toxin, might misdiagnose a patient with *C. difficile* carriage as a CDI case-patient and thereby result in overestimation of the CDI incidence (*5**,**6*). NAAT-alone testing is commonly used by the laboratories in the EIP surveillance sites (*4*); 47% of CDI cases identified in 2017 were diagnosed by a laboratory that used NAAT-alone testing (*7*). Conversely, SOC practices might fail to collect or appropriately test a stool specimen from a person with diarrhea and thereby underdiagnose CDI, which will result in underestimation of CDI incidence (*8**–**11*).

Incidence estimates are essential for evaluating the need for public health interventions aimed at reducing the CDI burden. We conducted a population-based study to determine CDI incidence and to evaluate the potential effect of misdiagnosis caused by SOC specimen collection and testing practices on CDI incidence estimates in Louisville, Kentucky, USA.

## Methods

### Study Design, Population, and Setting

Study staff conducted daily, prospective, active surveillance for incident diarrhea cases (>3 stools with Bristol scale >5 in previous 24 hours) among eligible inpatients (Louisville residents >50 years of age) by visiting inpatients, reviewing medical charts, and meeting with nursing staff. Surveillance was conducted on all 119 wards (including 26 intensive care units [ICUs]) at 8 of 9 Louisville adult hospitals from October 14, 2019, through October 13, 2020, with a surveillance pause during April 12, 2020‒August 16, 2020 because of hospital restrictions enacted in response to COVID-19. Participating hospitals had 84.4% (2,596/3,077) of the Louisville adult hospital beds. Population demographics of Louisville and the United States are similar (*12*).

### Study and SOC-Related Procedures

All eligible inpatients with incident diarrhea were invited to participate in the study. After written informed consent was obtained, a study stool specimen was collected by study staff from inpatients with diarrhea and screened by using C. Diff Quik Chek Complete, a rapid, membrane, enzyme-linked immunosorbent assay (Alere Techlab, https://www.techlab.com) (*13*). Before the COVID-19 pause, study stool specimens that were glutamate dehydrogenase (GDH)‒positive or GDH-negative/toxin-positive were sent to the Pfizer laboratory in Pearl River, New York, USA, for NAAT and, if NAAT positive, for automated cell cytotoxicity neutralization assay (CCNA) testing. CCNA testing at the Pfizer laboratory has been validated to provide specific, sensitive, and reproducible results to support CDI clinical and epidemiology studies (*14*).

After the pause, all study specimens were tested by Quik Chek and sent to the Pfizer laboratory for NAAT and CCNA testing. Shipped specimens were frozen on dry ice (replenished daily) and stored, upon receipt, in −80°C freezers. Throughout the study, patients with GDH-positive or GDH-negative/toxin-positive specimens were followed up by study staff for 90 days to determine outcomes, and SOC stool specimen collection by hospital staff occurred in parallel and independent of study procedures. For SOC CDI testing at the 8 participating hospitals, 2 used Quik Chek only, 5 used Quik Chek with NAAT of Quik Chek discordant specimens (but during the study, 1 changed to NAAT-alone testing and 1 to NAAT with toxin enzyme-linked immunosorbent assay testing of NAAT-positive), and 1 used NAAT-alone testing. Actual SOC test results (i.e., NAAT-positive or NAAT-negative) were provided to clinicians. For our analysis, we defined NAAT-positive/toxin-negative as SOC *C. difficile* carriage.

### Study Case Definition and Classification

A study CDI case-patient was a patient who had a NAAT-positive/CCNA-positive specimen or a patient who had a NAAT-positive specimen and pseudomembranous colitis (PMC). A CDI recurrent case was the occurrence of diarrhea <56 days after resolution of a previous case of diarrhea. A primary CDI case was a nonrecurrent CDI case. CDI in hospitalized case-patients was classified as hospital-onset (i.e., positive stool specimen collected >3 days after admission), community-onset (i.e., positive stool specimen collected in an outpatient setting or <3 days after admission), healthcare-associated (i.e., positive stool specimen in a person with hospital-onset or in a person with community-onset with a documented overnight stay in the 12 weeks before stool specimen collection), or community-associated (i.e., positive stool specimen in a person with community-onset with no documented overnight stay before the current admission in the 12 weeks before stool specimen collection) (*15*).

### Comparisons among Study and SOC Inpatients with Diarrhea

We reviewed medical records to compare the characteristics of inpatients with diarrhea who were enrolled and not enrolled in the study and to compare the characteristics of enrolled inpatients with diarrhea who had and did not have a study CDI test. We used χ^2^ tests (for categorical or binary variables) and t-tests (for continuous variables) to evaluate differences between the groups by using SAS Studio 3.71 (https://www.sas.com).

We compared laboratory results of study and SOC specimens collected from inpatients with diarrhea to determine the frequency of discordant results indicating SOC laboratory testing CDI overdiagnosis or underdiagnosis compared with study testing. We used laboratory results of specimens from inpatients with diarrhea who had a study specimen tested but did not have a SOC specimen collected to determine the frequency of SOC CDI underdiagnosis caused by lack of SOC specimen collection. We compared laboratory results of specimens collected after the pause from inpatients with diarrhea who had a study specimen screened by GDH and tested by NAAT to determine the negative predictive value (NPV) of the GDH test compared with the NAAT.

### Estimation of Population-Based Hospitalized CDI Incidence

We estimated population-based hospitalized CDI incidence among Louisville residents >50 years of age (n = 276,456), >65 years of age (n = 127,864), and >75 years of age (n = 51,509) after adjusting the number of study CDI cases for the percentage of Louisville adult hospital beds that were present in the nonparticipating hospital (multiplying by the inverse of the percentage of Louisville beds in participating hospitals [84.4%]) and for the percentage of inpatients with diarrhea who did not have a study specimen collected (multiplying by the inverse of percentage of participants with specimen collected [67.9%]). In a sensitivity analysis, we adjusted further the number of study CDI cases for specimens that were NAAT positive but with quantity not sufficient (QNS) for CCNA testing (multiplying by the inverse of the percentage of NAAT positive with CCNA testing [93.3%]) and, using the NPV of the GDH test compared with NAAT, for specimens that were not tested by NAAT (multiplying by the inverse of NPV [93.8%]).

### Ethics

This study was approved by the institutional review boards at each hospital. It was also conducted in accordance with the study protocol.

## Results

### Study and SOC Incident CDI Cases

**Figure 1:**
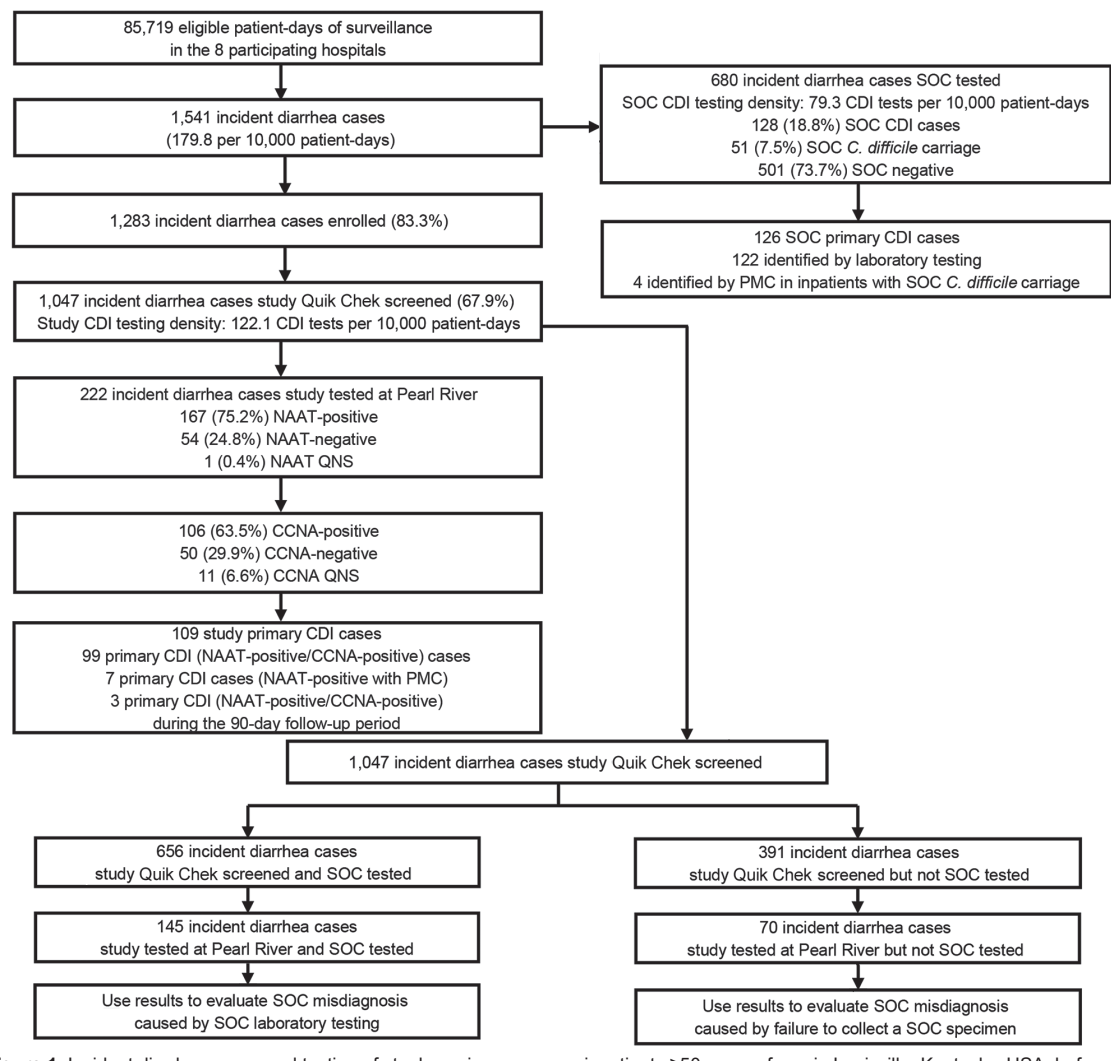
Incident diarrhea cases and testing of stool specimens among inpatients ≥50 years of age in Louisville, Kentucky, USA, before the COVID-19 pause in study of misdiagnosis of CDI by SOC specimen collection and testing among hospitalized adults, October 14, 2019–April 11, 2020. CCNA, cell culture cytotoxicity neutralization assay; CDI, *Clostridioides difficile* infection; NAAT, nucleic acid amplification test; PMC, pseudomembranous colitis; QNS, quantity not sufficient; Quik Chek, C. Diff Quik Chek Complete (Alere Techlab, https://www.techlab.com); SOC, standard-of-care.

**Figure 2:**
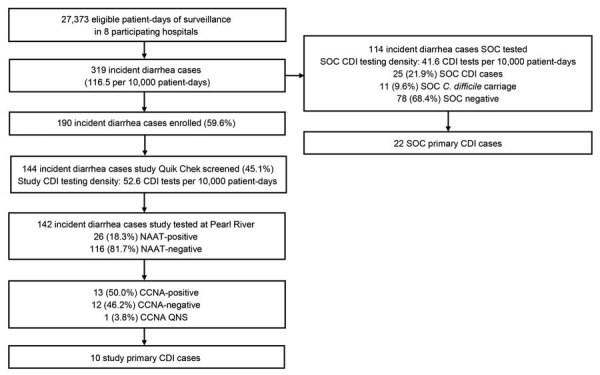
Incident diarrhea cases and testing of stool specimens among inpatients ≥50 years of age in Louisville, Kentucky, USA, after the COVID-19 pause in study of misdiagnosis of CDI by SOC specimen collection and testing among hospitalized adults, August 17, 2020–October 13, 2020. CCNA, cell culture cytotoxicity neutralization assay; CDI, *Clostridioides difficile* infection; NAAT, nucleic acid amplification test; QNS, quantity not sufficient; Quik Chek, C. Diff Quik Chek Complete (Alere Techlab, https://www.techlab.com); SOC, standard-of-care.

Study staff identified 1,541 incident diarrhea cases among 85,719 patient-days before the COVID-19 pause (Figure 1). SOC specimens were collected from 680 inpatients with diarrhea, for a SOC CDI testing density of 79.3/10,000 patient-days. Study specimens were collected from 1,047 (67.9%) inpatients with diarrhea, for a study CDI testing density of 122.1/10,000 patient-days; the study enrollment rate was 83.3% (1,283/1,541) and the specimen collection rate among enrolled participants was 81.6% (1,047/1,283) (Table 1). Patient transfer or discharge was the main reason that a specimen was not collected from all enrolled participants. Study staff identified 319 incident diarrhea cases among 27,373 patient-days after the COVID-19 pause. After the pause, study specimens were collected from 144 inpatients with diarrhea for a study CDI testing density of 52.6/10,000 patient-days; the study enrollment rate was 59.6% (190/319), and the specimen collection rate was 75.8% (144/190) (Figure 2).

**Table 1:** Hospital-based incidence of diarrhea, enrollment rate, stool collection rate, and CDI testing density in participating hospitals before the COVID-19 pause in study of misdiagnosis of CDI by SOC specimen collection and testing among hospitalized adults, Louisville, Kentucky, USA, October 14, 2019–April 11, 2020*

**Hospital†**	**No. adult hospital beds**	**Surveillance initiation date, 2019**	**No. eligible patient-days of surveillance**	**No. incident diarrhea cases identified**	**Diarrhea incidence‡**	**No. (%) incident diarrhea cases enrolled**	**No. (%) study stool specimens collected from enrolled cases**	**Study CDI stool specimen testing rate, %**	**Study CDI testing density§**
A	671	Oct 14	19,166	195	101.7	163 (83.6)	123 (75.5)	63.1	64.2
B	377	Oct 14	16,252	328	201.8	282 (86.0)	259 (91.8)	79.0	159.4
C	367	Oct 14	11,658	247	211.9	208 (84.2)	183 (88.0)	74.1	157.0
D	348	Oct 14	8,723	143	163.9	122 (85.3)	98 (80.3)	68.5	112.3
E	336	Nov 4	11817	281	237.8	218 (77.6)	133 (61.0)	47.3	112.5
F	200	Dec 23	4,256	41	96.3	32 (78.0)	21 (65.6)	51.2	49.3
G	170	Nov 4	5,754	146	253.7	120 (82.2)	98 (81.7)	67.1	170.3
H	127	Oct 14	8,093	160	197.7	138 (86.3)	132 (95.7)	82.5	163.1
Total	2,596	Oct 14–Dec 23	85,719	1,541	179.8	1,283 (83.3)	1,047 (81.6)	67.9	122.1

*CDI, *Clostridioides difficile* infection; SOC, standard-of-care. †Participating hospitals are listed in descending order of the number of adult hospital beds. ‡Cases per 10,000 patient-days. §Tests per 10,000 patient‑days.

Before the pause, 222 specimens were sent to the Pfizer laboratory after Quik Chek testing (74 GDH-positive/toxin-positive, 146 GDH-positive/toxin-negative, and 2 GDH-negative/toxin-positive); 163 (74.8%) were NAAT-positive. Of the NAAT-positive specimens, 103 (63.2%) were CCNA-positive, 49 (30.1%) were CCNA-negative, and 11 (6.7%) were QNS for CCNA testing. Of 103 NAAT-positive/CCNA-positive cases, 99 (96.1%) were primary CDI cases, and of the 49 NAAT-positive/CCNA-negative cases, 10 (20.4%) were primary CDI cases (7 had PMC and 3 had a specimen collected during the 90-day follow-up period that was NAAT-positive/CCNA-positive), for a total of 109 study-identified primary CDI cases. Before the pause, SOC testing identified 128 CDI cases, of which 126 were primary CDI cases (15 were identified by NAAT alone). After the pause, study testing identified 10 primary CDI cases, and SOC testing identified 22 primary CDI cases.

### CDI Misdiagnosis by SOC

An SOC specimen and a study specimen were collected and tested from 145 cases before the pause, of which 7 were recurrent CDI cases, yielding 138 for the evaluation of SOC misdiagnosis; of those cases, 75 (54.3%) were SOC primary CDI cases and 79 (57.2%) were study primary CDI cases (Table 2). Of 75 SOC primary CDI cases, 12 (16.0%) were not study-diagnosed as CDI cases; 6 NAAT-positive/CCNA-negative (SOC testing: 3 GDH-positive/toxin-positive/NAAT-positive and 3 NAAT-positive/toxin-positive) and 6 NAAT-negative (SOC testing: 3 GDH-positive/toxin-positive/NAAT-positive and 3 GDH-positive/toxin-positive). Of 79 study primary CDI cases, 16 (20.3%) were not SOC-diagnosed as CDI cases; SOC test results for those 16 were 13 NAAT-positive/toxin-negative, 2 NAAT-negative, and 1 GDH-negative/toxin-negative. In addition, 70 case-patients before the pause had a study specimen tested but without a SOC specimen collected; of those, 28 were study primary CDI case-patients. Therefore, 44 study-diagnosed primary CDI cases were not SOC diagnosed: 16 (36.4%) SOC-undiagnosed after SOC testing and 28 (63.6%) SOC-undiagnosed because of lack of specimen collection. Of 28 study-diagnosed CDI case-patients who were not SOC diagnosed because of lack of specimen collection, 11 (39.3%) were taking laxatives.

**Table 2:** SOC misdiagnosis caused by SOC laboratory testing after excluding recurrent CDI cases in study of misdiagnosis of CDI by SOC specimen collection and testing among hospitalized adults, Louisville, Kentucky, USA, October 14, 2019–April 11, 2020*

**SOC CDI test results**	**No. study primary CDI cases**	**No. not study primary CDI cases**	**Total**
SOC primary CDI cases	63	12	75
SOC *C. difficile* carriage	13	17	30
Not SOC primary CDI cases	3	30	33
Total	79	59	138
*CDI, *Clostridioides difficile* infection; SOC, standard-of-care.			

### Characteristics of Inpatients with Study-Diagnosed and SOC-Diagnosed CDI

The median age of the 109 study inpatients with primary CDI identified before the pause was 72 (range 50–98) years. A total of 63 (57.8%) were women. Based on the diarrhea onset date, 23 (21.1%) were community-onset and community associated and 86 (78.9%) were healthcare associated; of the healthcare-associated cases, 55 (64.0%) were community-onset and 31 (36.0%) were hospital-onset. Of the 109 study patients with primary CDI, 18 (16.5%) had PMC, 36 (33.0%) were admitted to an ICU, and 21 (19.3%) died within 90 days of CDI diagnosis. The median patient age among the CDI cases who died was 78 (range 56–95) years.

Characteristics of 44 study-diagnosed but SOC-undiagnosed inpatients who had primary CDI before the pause and the 126 SOC-diagnosed inpatients who had primary CDI before the pause were similar except that the SOC-undiagnosed inpatients who had CDI were more likely to be hospital-onset CDI case-patients and less likely to have PMC (Table 3). Of the 19 study-diagnosed but SOC-undiagnosed hospital-onset CDI cases, 16 (84%) were SOC-undiagnosed because an SOC specimen was not collected. On the basis of the 109 study-diagnosed primary CDI cases before the pause, SOC practices underdiagnosed 40.4% (44/109) of the study-diagnosed primary CDI cases. Because SOC overdiagnosed 16.0% of the study-diagnosed primary CDI cases and underdiagnosed 40.4% of study-diagnosed primary CDI cases, SOC testing identified 24.4% fewer primary CDI cases than did the study.

**Table 3:** Comparison between SOC-diagnosed primary CDI cases (n = 126) and study-diagnosed but not SOC-diagnosed primary CDI cases (n = 44) in study of misdiagnosis of CDI by SOC specimen collection and testing among hospitalized adults, Louisville, Kentucky, USA, October 14, 2019–April 11, 2020*

**Characteristic**	**SOC-diagnosed primary CDI cases, n =126**	**Study-diagnosed but not SOC-diagnosed primary CDI cases, n = 44†**	**p value**
Demographics			
Median age, y, (IQR)	73 (61‒81)	67 (59‒76)	0.13
Female sex	74 (58.7)	27 (61.4)	0.76
Signs and symptoms			
Fever	11 (9.1)	6 (13.6)	0.40
Nausea	38 (34.5)	11 (25.5)	0.29
Abdominal cramping	19 (19.2)	5 (12.8)	0.37
Dehydration	13 (12.5)	2 (5.3)	0.21
Location of diarrhea onset			
Hospital-onset‡	22 (17.5)	19 (43.2)	<0.01
Medical history			
Dementia	14 (11.1)	4 (9.1)	0.71
Cancer	42 (33.3)	11 (25.0)	0.30
Congestive heart disease	41 (32.5)	23 (52.3)	0.02
Pneumonia	33 (26.2)	13 (29.5)	0.67
Inflammatory bowel disease	17 (13.5)	3 (6.8)	0.24
Diabetes	47 (37.3)	21 (47.7)	0.22
Medical course			
Recurrence	4 (3.2)	0 (0)	0.23
PMC	26 (20.8)	0 (0)	<0.01
Transferred to ICU	36 (28.6)	18 (40.9)	0.13
Death at 90 days	20 (15.9)	7 (15.9)	1.00

*Values are no. (%) except as indicated. CDI, *Clostridioides difficile* infection; ICU, intensive care unit; IQR, interquartile range; PMC, pseudomembranous colitis; SOC, standard-of-care. †Includes 28 study-diagnosed primary CDI cases that did not have SOC testing and 16 study-diagnosed primary CDI cases that had SOC testing but were not SOC-diagnosed primary CDI cases. ‡Defined as onset of diarrhea ≥3 days after hospital admission.

After the pause, study specimens from 142 cases were screened by Quik Chek and tested by NAAT; of the 113 GDH-negative/toxin-negative specimens, 106 (93.8%) were NAAT-negative. Therefore, the NPV of the Quik Chek GDH test compared with NAAT was 93.8%, indicating that an estimated 6.2% of NAAT-positive specimens might have been missed because of the screening used during the pre‒COVID-19 period.

### Comparisons among Study and SOC Inpatients with Diarrhea

A review of the medical records of inpatients with diarrhea before the pause (Table 4) indicated that patients who were enrolled and those not enrolled had similar demographics and medical histories, except enrolled patients were more likely to be women and less likely to die in the 90 days after diarrhea onset. Among diarrhea case-patients who were enrolled (Table 5), patients with and without a stool specimen collected for CDI testing had similar demographics and medical histories, except that those who had a stool specimen collected were less likely to be Black and more likely to have taken antimicrobial drugs in the previous 3 months.

**Table 4:** Comparison between enrolled and nonenrolled incident diarrhea cases before the COVID-19 pause (n = 1,490) in study of misdiagnosis of CDI by SOC specimen collection and testing among hospitalized adults, Louisville, Kentucky, USA, October 14, 2019–April 11, 2020*

**Characteristic**	**Enrolled, n =1,249**	**Nonenrolled, n = 241**	**p value**
Demographics			
Median age, y	68	68	NA
Female sex	737 (59)	119 (49)	<0.01
Black	297 (24)	62 (26)	0.52
Medical history			
Heart disease	638 (51)	131 (54)	0.35
Cancer	371 (30)	57 (24)	0.06
Stroke	235 (19)	41 (17)	0.51
Dementia	85 (7)	27 (11)	0.02
Antimicrobial drug use in past 2 weeks	370 (30)	74 (31)	0.74
Admission diagnosis			
Heart disease	131 (10)	26 (11)	0.89
Respiratory disease	105 (8)	21 (9)	0.88
Pneumonia	95 (8)	20 (8)	0.71
Nervous system disease	61 (5)	21 (9)	0.02
Medical course			
Transferred to ICU	297 (24)	73 (30)	0.03
Death in 90 days	152 (12)	53 (22)	<0.01

*Values are no. (%) except as indicated. Surveillance ascertained 1,541 incident diarrhea cases. Medical record review was conducted at 7 of 8 participating hospitals. Hospital F, where 41 incident diarrhea cases were ascertained, did not participate in medical record review. Data from 10 medical records at hospitals A–E, G, and H were not available for this analysis. Data are presented in descending order, where applicable, of Enrolled column. CDI, *Clostridioides difficile* infection; ICU intensive care unit; NA, not applicable; SOC, standard-of-care.

**Table 5:** Comparison between inpatients who had a stool specimen collected for CDI testing and inpatients who did not among incident diarrhea cases enrolled before COVID-19 pause (n = 1,283) in study of misdiagnosis of CDI by SOC specimen collection and testing among hospitalized adults, Louisville, Kentucky, USA, October 14, 2019–April 11, 2020*

Characteristic	Study stool specimen collected, n = 1,047	Study stool specimen not collected, n = 236	p value
Demographics			
Median age, y (IQR)	68 (61–78)	67.5 (60–77)	0.46
Female sex	595 (56.8)	146 (61.9)	0.16
Black	218 (21.5)	81 (34.8)	<0.01
Hispanic	18 (1.8)	2 (0.9)	0.33
Signs and symptoms			
Abdominal pain	283 (28.7)	53 (23.8)	0.14
Fever	109 (10.7)	18 (7.8)	0.18
Blood or pus in stool	90 (9.3)	20 (9.0)	0.91
Medical history			
Overnight stay in healthcare facility in previous 12 weeks	615 (60)	150 (63.8)	0.39
Diabetes	432 (41.5)	91 (38.9)	0.46
Antimicrobial drug use in previous 3 months	387 (39.2)	63 (26.9)	< 0.01
Cancer	313 (30.2)	59 (25.1)	0.12
Congestive heart disease	305 (29.3)	64 (27.2)	0.53
CDI in past 5 years	66 (7.5)	18 (8.1)	0.76
Dementia	76 (7.3)	14 (5.9)	0.46
Inflammatory bowel disease	65 (6.3)	10 (4.3)	0.26
Hemiplegia/quadriplegia	54 (5.2)	8 (3.4)	0.25

*Values are no. (%) except as indicated. Data are presented in descending order of Study stool specimen collected column. Values of missing or unknown were excluded from percentage and p value calculations. CDI, *Clostridioides difficile* infection; IQR, interquartile range; SOC, standard-of-care.

### Population-Based Hospitalized CDI Incidence

The study incidence was 154 hospitalized CDI cases/100,000 population/year and the SOC incidence was 121 hospitalized CDI cases/100,000 population/year for persons >50 years of age before the pause. The study hospitalized CDI incidence was 226/100,000 population/year for persons >65 years of age and 334/100,000 population/year for persons >75 years of age. In the sensitivity analysis adjusted for CCNA QNS and for stool specimens that were not NAAT/CCNA tested because of the GDH screen, the study incidence was 202 hospitalized CDI cases/100,000 population/year for persons >50 years of age, 296 hospitalized CDI cases/100,000 population/year for persons >65 years of age, and 438 hospitalized CDI cases/100,000 population/year for persons >75 years of age.

## Discussion

In a comprehensive population-based surveillance study of diarrhea among hospitalized patients in the United States, including a high stool specimen CDI testing density and rigorous laboratory testing, we identified high incidence of CDI among hospitalized persons >50 years of age, with frequent severe clinical consequences. Among inpatients hospitalized with primary CDI identified before the COVID-19 pandemic, almost one fifth had PMC, one third were admitted to an ICU, and one fifth died in the 90 days after diagnosis. When adjusted for the diarrhea case-patients without a study CDI test result, the incidence was 202 hospitalized primary CDI cases per 100,000 persons >50 years of age per year, which is 44% higher than the incidence of 140 hospitalized CDI cases per 100,000 persons >50 years of age per year that were reported in the CDC EIP CDI surveillance system in 2019 (*4*). The study also found 24.4% more hospitalized primary CDI case-patients than were found by independent SOC CDI testing of the same diarrhea cases.

Public health surveillance systems, including EIP surveillance, rely on SOC CDI stool collection and testing practices. However, results from this study indicate that public health surveillance systems probably underestimate the incidence of hospitalized patients who have CDI. There are 112 million persons >50 years of age in the United States, and results from our study indicate that at least 226,000 persons in that age group are hospitalized with CDI each year in the United States, rather than the 160,000 estimated by EIP surveillance (*4*).

The effect of SOC specimen collection and testing practices on CDI incidence has been frequently discussed (*5**,**6**,**8**–**11*). SOC testing can result in overdiagnosis of CDI and overestimation of CDI incidence, if SOC testing practices misidentify *C. difficile* carriage as CDI. Concerns have been raised about use of NAAT alone for CDI diagnosis because a NAAT-positive specimen only indicates the presence of toxigenic *C. difficile* and does not identify the presence of toxin (*5**,**6*). Those concerns have led to guidance in Europe to use 2-step testing with the first step determining the presence of toxin, the toxin gene, or *C. difficile* and positive specimens tested in the second step so that results of the 2 steps confirm the presence of *C. difficile* and the presence of free toxin (*16**,**17*).

NAAT alone is commonly used in clinical laboratories in the United States (*4*,*7*), where the guidance supports use of NAAT alone if there are institutional criteria for patient stool submission (*18*). The frequent use of NAAT alone in the United States means that CDI incidence could be overestimated. However, results from this study indicate that SOC CDI testing practices, when compared with comprehensive and rigorous NAAT and CCNA testing, result in CDI overdiagnosis less frequently than CDI underdiagnosis. Therefore, CDI overdiagnosis caused by SOC practices is unlikely to result in overestimation of CDI incidence in public health surveillance in the United States.

SOC CDI underdiagnosis can result from either misdiagnosis by laboratory testing or lack of specimen collection. Of the instances of SOC CDI underdiagnosis in our study, 36.4% occurred when study testing diagnosed CDI but SOC testing did not, and 63.6% occurred when SOC did not collect a specimen. Perhaps not surprisingly, because guidelines recommend against CDI testing of patients who had received a laxative within the preceding 48 hours (*18*), a high percentage (39.3%) of the study-diagnosed CDI case-patients who were not SOC-diagnosed because of lack of specimen collection were receiving laxatives. We found that SOC-diagnosed CDI cases were less likely to be hospital-onset cases than study-diagnosed (but SOC-undiagnosed) cases, perhaps reflecting less willingness to diagnosed hospital-onset CDI, which might be associated with penalties for having an increased incidence of hospital-onset CDI cases.

We also found that a higher percentage of study-identified primary CDI cases were healthcare-associated cases (78.9%) than as reported in EIP surveillance (48%) during 2019 (*4*), a difference that is, in part, related to the higher percentage of hospital-onset cases in our study. Other investigators have reported on CDI underdiagnosis from not collecting specimens and the resulting effect on CDI incidence estimates (*8**–**11*). Because clinical judgment can identify patients with diarrhea who are more likely to have CDI, clinical judgment should be the most essential factor in deciding when to collect a specimen from a patient with diarrhea. The objective of this study was not to suggest when CDI testing is appropriate but to illuminate that CDI underdiagnosis probably results in underestimation of the CDI incidence in public health surveillance in the United States.

The strength of this study was that we conducted active surveillance for incident diarrhea cases and collected specimens from inpatients with diarrhea, in parallel with (and independent of) SOC specimen collection and testing. Surveillance, patient informed consent, and study specimen collection were conducted by designated study staff, independent of hospital staff, to reduce the influence on SOC practices. The extent to which the study influenced SOC practices is unknown, but the study might have increased awareness of CDI and increased SOC CDI testing. Use of the independent study staff resulted in high enrollment and specimen collection rates, which resulted in a small percentage of inpatients with diarrhea not having a study specimen collected for CDI testing. Another strength of this population-based study was that it was conducted in Louisville, Kentucky, which has been shown to be representative of the United States (*12*).

Surveillance for the study was interrupted after 6 months because of hospital-enacted COVID-19 restrictions. After a 4-month pause, surveillance was reinstated, but access to patients remained restricted, which resulted in a decrease in the study enrollment rate. After reinstatement of surveillance, most of the hospitalized patients were admitted for treatment of COVID-19, resulting in a different inpatient population than that for the prepandemic period. Therefore, we restricted our analysis estimating the population-based hospitalized CDI incidence and the extent of SOC CDI misdiagnosis to the data collected before the COVID-19 pause. The only data collected after the pause that were used in the incidence estimates were for the determination of the NPV of the GDH test compared with the NAAT, an evaluation that was unlikely to be affected by differences in the inpatient populations.

Limitations of this study include that, despite the intense active surveillance, incident diarrhea cases might have been undetected, and the incidence estimates are based only on 6 months of surveillance. Although minor seasonal variation of hospitalized CDI incidence has been reported in the United States (*19*), there was limited variation in the monthly CDI incidence during this study (data not shown). A study limitation is that, when assessing CDI misdiagnosis by SOC testing, study and SOC specimen collection were independent, and therefore the same specimen was not tested by both methods; this limitation is most evident with SOC overdiagnosed cases, some of which might have been CDI cases. Another study limitation is that a specimen was not available for study CDI testing from all inpatients with diarrhea for various reasons.

Furthermore, insufficient stool was available for CCNA testing for some of the study specimens collected. Another reason was that the NPV of the GDH screening test indicates that the GDH test probably identified a small number of primary CDI cases incorrectly as GDH-negative. Because a comparison of the medical records of the diarrhea case-patients with and without study CDI testing demonstrated few differences, we conducted a sensitivity analysis to evaluate the effect of not conducting CDI testing for all inpatients with diarrhea, adjusting the incidence estimates for inpatients with diarrhea who did not have a study CDI test. Finally, there is no recognized standard laboratory test for CDI diagnosis. Although we used a validated automated CCNA test, study laboratory testing might have missed CDI cases (*20*), emphasizing that the diagnosis of CDI should not be based on laboratory results alone.

In conclusion, we identified a high population-based incidence of hospitalized CDI case-patients that had frequent severe clinical consequences in Louisville, Kentucky, which, when generalized nationwide, demonstrates that the hospitalized CDI burden is high in the United States. Furthermore, the hospitalized CDI burden in the United States is probably higher than is currently reported in public health surveillance systems because of CDI underdiagnosis by SOC specimen collection and testing practices. This high burden in the United States indicates that additional interventions are needed for the prevention of CDI.
